# S-layer and cytoplasmic membrane – exceptions from the typical archaeal cell wall with a focus on double membranes

**DOI:** 10.3389/fmicb.2014.00624

**Published:** 2014-11-25

**Authors:** Andreas Klingl

**Affiliations:** Plant Development, Department of Biology, Biocenter LMU Munich – Botany, Ludwig Maximilian University MunichMunich, Germany

**Keywords:** archaea, S-layer, outer membrane, cytoplasmic membrane, cell wall

## Abstract

The common idea of typical cell wall architecture in archaea consists of a pseudo-crystalline proteinaceous surface layer (S-layer), situated upon the cytoplasmic membrane. This is true for the majority of described archaea, hitherto. Within the crenarchaea, the S-layer often represents the only cell wall component, but there are various exceptions from this wall architecture. Beside (glycosylated) S-layers in (hyper)thermophilic cren- and euryarchaea as well as halophilic archaea, one can find a great variety of other cell wall structures like proteoglycan-like S-layers (Halobacteria), glutaminylglycan (Natronococci), methanochondroitin (*Methanosarcina*) or double layered cell walls with pseudomurein (*Methanothermus* and *Methanopyrus*). The presence of an outermost cellular membrane in the crenarchaeal species *Ignicoccus hospitalis* already gave indications for an outer membrane similar to Gram-negative bacteria. Although there is just limited data concerning their biochemistry and ultrastructure, recent studies on the euryarchaeal methanogen *Methanomassiliicoccus luminyensis*, cells of the ARMAN group, and the SM1 euryarchaeon delivered further examples for this exceptional cell envelope type consisting of two membranes.

## INTRODUCTION

Microorganisms and especially archaea can be found in almost any kind of extreme environment, although they are not limited to them: high temperature, high acidity, high pressure, anoxic, no organic substrates. In those habitats, various species of hyperthermophilic or more generally extremophilic archaea were found and described. Therefore, the general cell plan of the majority of these extremophilic archaea and especially their cell wall architecture might represent the most basic and archaic version: a pseudo-crystalline proteinaceous surface layer (S-layer), a so called S-layer which is situated upon a single cytoplasmic membrane which is enclosing the cytoplasm. This simple cell plan was found to be present in the majority of described archaeal species. Because of its simplicity and widespread distribution within the major groups of archaea and bacteria, it was already stated by Albers and Meyer (2011) that the S-layer might be the cell wall variant that has evolved the earliest. Especially within the crenarchaea, the S-layer usually depicts the only cell wall component. S-layer glycoproteins were first discovered and extensively studied in halophilic archaea, namely *Halobacterium salinarum* as well as in *Haloferax volcanii* (Houwink, 1956; Mescher and Strominger, 1976a,b; Lechner and Sumper, 1987; Sumper and Wieland, 1995; Sumper et al., 1990) and *Halococcus* (Brown and Cho, 1970) or methanogens like *Methanosarcina* (Kandler and Hippe, 1977), *Methanothermus fervidus* (Kandler and König, 1993; Kärcher et al., 1993) and *Methanococcus* species like *Methanococcus vannielii* and *Methanococcus thermolithotrophicus* (Koval and Jarrell, 1987; Nußer and König, 1987). Amongst others, several studies were carried out focusing on the S-layer in various *Sulfolobus* species. The members of the order Sulfolobales, e.g., *Sulfolobus solfataricus* or *Metallosphaera sedula*, represent model organisms for the basic structure of this kind of cell wall (Veith et al., 2009; Albers and Meyer, 2011).

But as various examples in the past could show, the archaeal cell wall architecture is not always that simple. Beside the (glycosylated) S-layers in halophilic, thermophilic and hyperthermophilic eury – as well as crenarchaea, one can find a great variety of totally different cell wall structures that sometimes resemble biological substances also found in eukaryotes and bacteria, e.g., glutaminylglycan in Natronococci, methanochondroitin in *Methanosarcina* or double layered cell walls containing pseudomurein in *Methanothermus* and *Methanopyrus* to name just a few (König et al., 2007; Albers and Meyer, 2011; Klingl et al., 2013).

In addition, the finding of an energized outermost cellular membrane in the well described *Ignicoccus hospitalis* and related species already indicated the possibility of an outer membrane (OM), as it is present in Gram-negative bacteria. Furthermore, recent results on the SM1 euryarchaeon, ultra-small ARMAN cells and *Methanomassiliicoccus luminyensis* strengthened the idea of a real archaeal OM and, besides others, will also be discussed here (Comolli et al., 2009; Dridi et al., 2012; Perras et al., 2014). And in this concern, the possible functions of an OM in regard to the bacterial version as well as challenges concerning energetic problems become apparent.

## ARCHAEAL CELL WALLS

**FIGURE 1:**
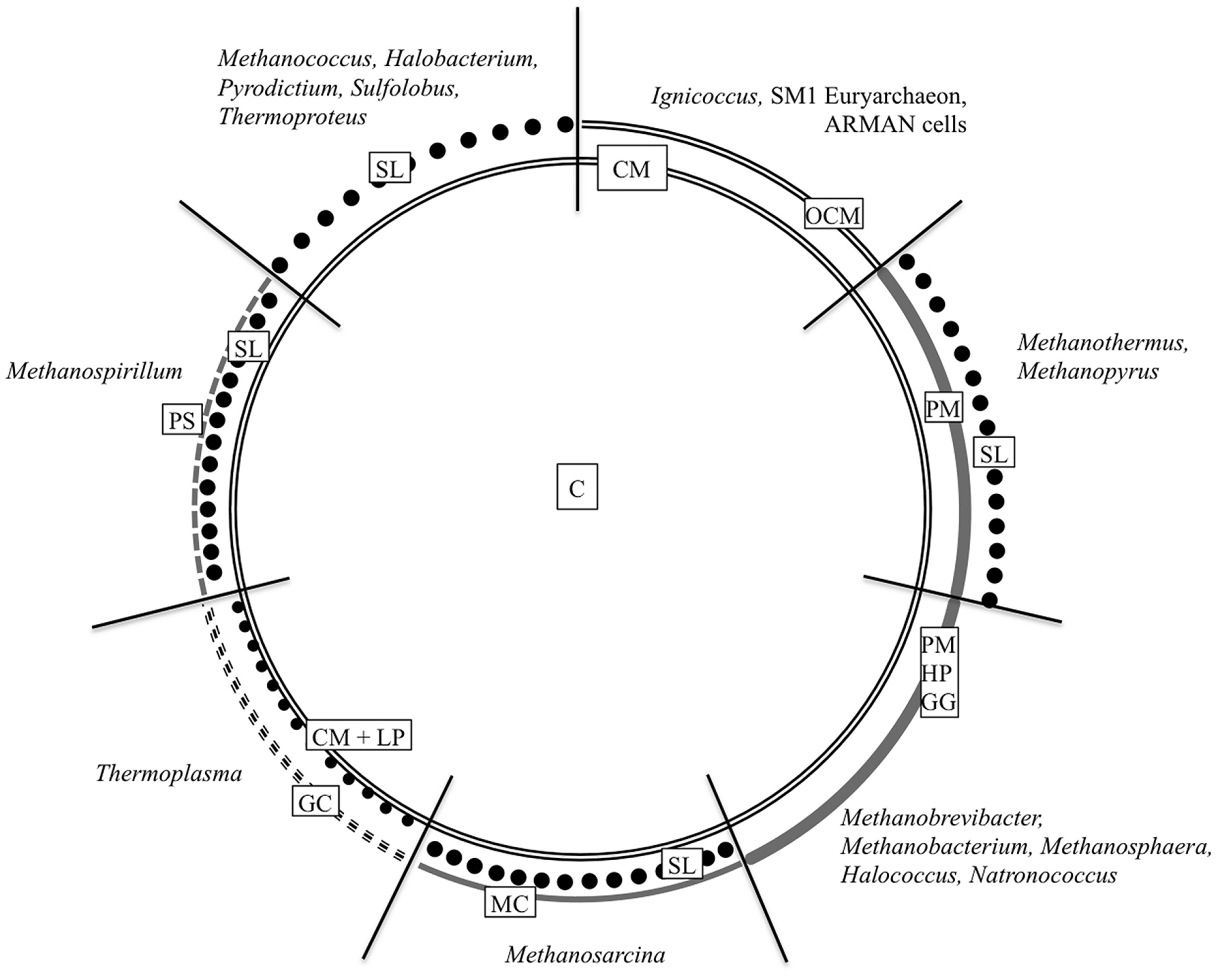
**Cell wall organization of Archaea.** The schematic illustration summarizes the most common archaeal cell wall types including the most relevant genera. C, cytoplasm; CM, cytoplasmic membrane; GC, glycocalyx; GG, glutaminylglycan; HP, heteropolysaccharide; LP, lipoglycans; MC, methanochondroitin; OCM, outermost cellular membrane or outer membrane; PM, pseudomurein; PS, protein sheath; SL, S-layer. Based on König et al. (2007) ASM Press, Washington, DC, USA.

Similar to bacteria, the cytoplasm in archaea is enclosed by a cytoplasmic membrane built up mainly of glycerol phosphate phospholipids, although with slight differences in membrane lipid composition (Kates, 1992; Albers and Meyer, 2011; Klingl et al., 2013). But instead of fatty acids linked to the (*sn*)-1,2 positions of glycerol via ester bonds, the lipid core of archaea consists of C_5_ isoprenoid units coupled to glycerol via ether bonds in an archaea specific (*sn*)-2,3 position (Kates, 1978; Kates, 1992; Albers and Meyer, 2011). But this will not be discussed in here, as the main focus of this overview will be on the archaeal cell wall, especially on components, which are situated outside the cytoplasmic membrane. Most commonly, this cell wall is represented by a proteinaceous S-layer. But as the following overview will show, there are a lot of other cell wall variants (**Figure 1**). According to some recent findings, there will be a special focus on archaea that could be shown to be surrounded by double membranes.

### S-LAYER

**FIGURE 2:**
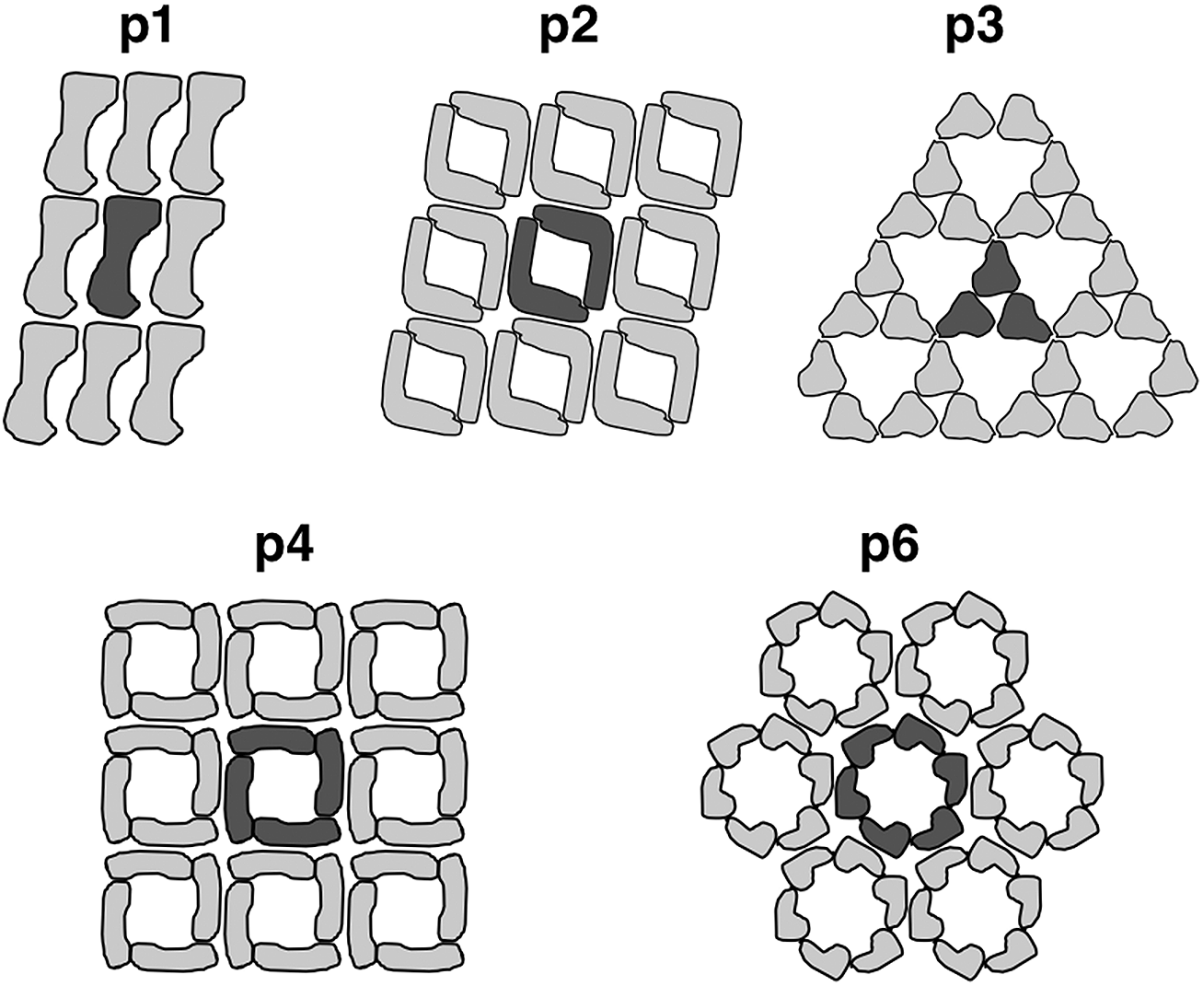
**Arrangement of S-layer subunits in the respective symmetry types.** The unit cell of the 2D-protein crystals are indicated in dark grey. Reproduced from Sleytr et al. (1999) with the permission of WILEY-VCH Verlag GmbH, Weinheim, Germany

Most commonly, the archaeal cell envelope consists of a protein or glycoprotein S-layer, a so called S-layer, forming a 2-D pseudo-crystalline array on the cell surface with a distinct symmetry (Kandler and König, 1985; Beveridge and Graham, 1991; Baumeister and Lembcke, 1992; Messner and Sleytr, 1992; Kandler and König, 1993; Sumper and Wieland, 1995; Veith et al., 2009; Albers and Meyer, 2011; Klingl et al., 2013). They are usually composed of one type of (glyco-)protein forming a central crystal unit consisting of two, three, four, or six subunits which equates p2-, p3-, p4- or p6-symmetry, respectively (**Figure 2**; Sleytr et al., 1988; Sleytr et al., 1999; Eichler, 2003).

This protein array is usually anchored in the cytoplasmic membrane via stalk like structures forming a quasi-periplasmic space. The lattice constants for those S-layer crystals were shown to vary between 11 and 30 nm with protein masses between 40 and 325 kDa (Messner and Sleytr, 1992; König et al., 2007). With some limitations, the S-layer symmetry as well as the center-to-center spacing can be used as a taxonomic trait (König et al., 2007; Klingl et al., 2011). For example, all S-layer proteins of *Sulfolobus* species described so far revealed a very rare p3-symmetry and a spacing around 21 nm (König et al., 2007; Veith et al., 2009). This symmetry was thought to be unique for the Sulfolobales until recent findings concerning the S-layer of *Nitrososphaera viennensis* could show that this member of the phylum Thaumarchaeota also has a surface protein with p3-symmetry (Stieglmeier et al., 2014).

The S-layer protein of *Halobacterium salinarum* was not only the first glycoprotein discovered in prokaryotes but also exemplifies the fact that S-layers are often highly glycosylated (Mescher and Strominger, 1976a,b; Kandler and König, 1998; König et al., 2007; Veith et al., 2009; Albers and Meyer, 2011). The glycosylation of halophilic S-layer proteins increases protein stability and also prevents degradation (Yurist-Doutsch et al., 2008). Besides the situation in halophilic archaea, the glycosylation may also contribute to a thermal stabilization of S-layer proteins as mentioned in Jarrell et al. (2014).

Concerning the potential function of S-layer proteins, several possibilities have been discussed (Engelhardt, 2007a,b): protection against high temperature, salinity (osmoprotection), low pH and maintenance of cell shape (exoskeleton). Especially within the crenarchaea, they comprise high temperature stability as they have to withstand temperatures around 80°C and pH below 2 in case of Sulfolobales (e.g., Veith et al., 2009). Herein, a high portion of charged amino acids as well as ionic interactions may play an important role (Haney et al., 1999). Another example for the high stability of S-layer proteins was shown for *Thermoproteus tenax* and *Thermofilum pendens*, where the rigid S-layer sacculus even withstands treatment with 2% SDS at 100°C for 30 min (König and Stetter, 1986; Wildhaber and Baumeister, 1987; König et al., 2007). In most euryarchaeota, the situation is totally different with highly labile S-layer proteins (e.g., *Archaeoglobus fulgidus*, König et al., 2007), which also makes it difficult to isolate the proteins. An exception from these findings is the S-layer of *Picrophilus*, which may be a side effect of its high acid stability.

For additional information on general properties of S-layer proteins, their genetic background and characteristic features, the reader’s attention should be pointed to some general reviews on this topic (e.g., Claus et al., 2001, 2005; König et al., 2007; Albers and Meyer, 2011). Furthermore, there are also several more focused studies on S-layer proteins of mesophilic and extremely thermophilic archaea (Claus et al., 2002) as well as mesophilic, thermophilic and extremely thermophilic methanococci (Akça et al., 2002).

### PSEUDOMUREIN, METHANOCHONDROITIN, AND PROTEIN SHEATHS

Furthermore, pseudomurein, a polymer which maintains the cell shape and perhaps also protects the cells, can be found as an additional second cell wall compound in all species of *Methanothermus* and *Methanopyrus* (König et al., 2007). It shows similarity to bacterial peptidoglycan but usually consists of *L*-*N*-acetyltalosaminuronic acid with a β-1,3 linkage to *D*-*N*-acetylglucosamine instead of *N*-acetylmuramic acid linked β-1,4 to *D*-*N*-acetylglucosamine as it is the case in bacterial murein (peptidoglycan). In addition, the crosslinking amino acids in pseudomurein are represented by L-amino acids (glutamic acid, alanine, lysine) instead of D-amino acids in murein (Kandler and König, 1993; König et al., 1994; Albers and Meyer, 2011).

In contrast to single cells, aggregates of *Methanosarcina spp.* produce a substance called methanochondroitin covering the S-layer with the latter one also being present in single cells (Kreisl and Kandler, 1986; Albers and Meyer, 2011). Methanochondroitin, which is similar to chondroitin in the connecting tissue of vertebrates (Kjellen and Lindahl, 1991), consists of a repeating trimer of two *N*-acetylgalactosamines and one glucuronic acid but differing from vertebral chondroitin in the molar ratio of the monomers and the fact that it is not sulfated (Albers and Meyer, 2011).

The methanogenic archaeal species *Methanospirillum hungatei* and *Methanosaeta concilii* form long chains that are surrounded by a proteinaceous sheath (Zeikus and Bowen, 1975). Beside its high stability against proteases and detergents, it also revealed a paracrystalline structure and functioning as a micro sieve (Kandler and König, 1978; Sprott and McKellar, 1980; König et al., 2007). The specialty of this sheath is that it is surrounding the whole chain and not just the single cells. Each cell is surrounded separately by an inner cell wall consisting of an S-layer (*Methanospirillum hungatei*) or an amorphous granular layer (*Methanosaeta concilii*; Zeikus and Bowen, 1975; Sprott et al., 1979; Zehnder et al., 1980; Beveridge et al., 1985, 1986; Shaw et al., 1985; Beveridge and Graham, 1991; Firtel et al., 1993; Albers and Meyer, 2011).

### GLUTAMINYLGLYCAN AND HALOMUCIN

In similarity to poly-γ-D-glutamyl polymers in *Bacillus*, *Sporosarcina* and *Planococcus*, such polymers were also found within the genus *Natronococcus* (Niemetz et al., 1997). In *Natronococcus occultus*, polyglutamin is forming the cell wall but in contrast to similar polymers found in bacteria, the wall polymer in this archaeum is glycosylated. It is consisting of approximately 60 monomers, which are linked via the γ–carboxylic group (König et al., 2007).

In the square shaped extremely halophilic euryarchaeon *Haloquadratum walsbyi*, cells are surrounded by an S-layer upon the cytoplasmic membrane. Depending on the strain C23^T^ or HBSQ001, the cells of *H. walsbyi* are surrounded by one or, even more complex, two S-layers, respectively (Burns et al., 2007). In addition, another protein called halomucin is present which is highly similar to mammalian mucin and probably helps the cells to thrive under conditions of up to 2 M MgCl_2_ (Bolhuis et al., 2006). Because of the presence of respective genes, *M. walsbyi* is most likely also surrounded by a poly-γ-glutamate capsule (Bolhuis et al., 2006; Albers and Meyer, 2011).

### TWO LAYERED CELL WALLS

For both *Methanothermus fervidus* and *Methanopyrus kandleri*, a cell envelope consisting of two distinct layers has been described (Stetter et al., 1981; Kurr et al., 1991; König et al., 2007). In the former case, it is formed by a pseudomurein layer (thickness 15–20 nm) covered by an external S-layer glycoprotein with p6-symmetry. In the latter case, the situation is similar except that no regular arrangement of the outermost layer could be shown for *Methanopyrus* (König et al., 2007). At this point it has to be mentioned that two layered cell walls are not just limited to *Methanothermus fervidus* and *Methanopyrus kandleri* because other archaea can also possess two cell walls, e.g., *Methanosarcina* species are covered with an S-layer and an optional layer of methanochondroitin. Another example is the previously mentioned *H*. *walsbyi* strain HBSQ001 that is covered by two S-layers.

### DOUBLE MEMBRANES

There are just a few examples of archaeal species described so far, which do not possess one of the previously mentioned cell wall polymers and structures. Members of the Thermoplasmatales like *Ferroplasma acidophilum* completely lack a cell wall, despite growing under harsh conditions like elevated temperatures and low pH. It is therefore thought that a glycocalyx, lipoglycans, or membrane-associated glycoproteins substitute the function of a cell wall for these organisms (Albers and Meyer, 2011). The hyperthermophilic sulfur-oxidizing crenarchaeal species *Ignicoccus hospitalis* was the first archaeon, for which a double membrane system was described (Huber et al., 2002, 2012; Rachel et al., 2002; Näther and Rachel, 2004; Junglas et al., 2008; Küper et al., 2010). This is also true for all other species within the genus *Ignicoccus* investigated up to date. It is a highly complex and dynamic system leading to a compartmentalized cell with a huge periplasm enclosed by both membranes. The width of this periplasm can vary from 20 up to 1000 nm (König et al., 2007; Huber et al., 2012). There are some clear differences between both membranes. The inner membrane (IM) consists of archaeol as well as caldarchaeol with the latter one forming tetraether lipids and therefore cannot be separated in freeze fracturing experiments (Rachel et al., 2002, 2010; Burghardt et al., 2007; Huber et al., 2012; Klingl et al., 2013) while the outermost cellular membrane contains archaeol. In addition, most of the polar head groups are glycosylated (Jahn et al., 2004). Interestingly, the ATP synthase as well as the S^0^-H oxidoreductase were shown to be located in this outermost membrane and not in the cytoplasmic membrane, as it could have been expected; *Ignicoccus hospitalis* therefore exhibits an energized outer cellular membrane (Küper et al., 2010).

Beside the two membranes of *Ignicoccus hospitalis* and other closely related species of the genus *Ignicoccus*, recent studies on other archaea could also confirm a double membrane system on these organisms. Three-dimensional cryo electron tomography on cells of some ultra-small archaea belonging to the philogenetically deeply branching and uncultivated ARMAN lineage revealed an inner and an OM enclosing a periplasm (Comolli et al., 2009). In this special case, they also found indications for cytochromes in the IM. During a study attempting to isolate human-associated archaea, a new genus named *Methanomassiliicoccus luminyensis* was described (Dridi et al., 2012). Although the quality of data concerning the ultrastructure of this organism was poor, it was still possible to recognize an electron dense layer outside the cytoplasmic membrane, most likely represented by an OM. The thick transparent layer mentioned in this study might depict the periplasm of *Methanomassiliicoccus luminyensis*. In a recent study concerning the ultrastructure of the cold-loving archaeal isolate SM1, an outer cellular membrane in addition to the cytoplasmic membrane could be documented as well (Perras et al., 2014).

With a second, outermost membrane, you get at least two separated compartments like in Gram-negative bacteria: the cytoplasm and the (pseudo)periplasm (Rigel and Silhavy, 2012). In Gram-negative bacteria, the periplasm can make up about 10% of the cell volume and constitutes an oxidizing environment, containing soluble proteins, the thin peptidoglycan layer and usually no ATP (Ruiz et al., 2006). In the special case of *Ignicoccus*, the volume of the intermembrane compartment as an analog to the bacterial periplasm can even be higher than that of the cytoplasm (Küper et al., 2010). Like in bacteria, the presence of membrane proteins and pores makes the OM a permeable and selective barrier (Rigel and Silhavy, 2012). Although there are differences in lipid and protein composition of the inner and outermost cellular membrane in *Ignicoccus hospitalis* (Burghardt et al., 2007; Küper et al., 2010) it still has to be elucidated if there is also an asymmetric OM containing LPS (lipopolysaccharide) present in archaea. In Gram-negative bacteria, one can find a phospholipid bilayer (IM) and usually an asymmetric bilayer in case of the OM, including proteins like transporters or channels (Ruiz et al., 2006; Rigel and Silhavy, 2012). In the OM, the inner leaflet is composed of phospholipids; the outer leaflet is mainly composed of LPS, which is essential for the barrier function of the OM (Ruiz et al., 2006): lipid A, a core oligosaccharide and an O-antigen polysaccharide with variations in length. In similarity to Gram-negative bacteria, archaea with two membranes are featuring several problems: They need lipoproteins and integral OM proteins (OMPs) in the OM. The latter ones are essential for intake of nutrients and export of waste products as they serve as channels (Ruiz et al., 2006). Furthermore, it also shows the importance of a specific system for the biogenesis of OMs and the secretion system in archaea as it was described for *Escherichia coli*, for example (Tokuda, 2009).

## SUMMARY AND OUTLOOK

Although a cytoplasmic membrane superimposed by an S-layer depicts the most common cell wall architecture in archaea, there are various other cell wall versions present in cren- as well as in euryarchaeota. As they were isolated from totally different biotopes, it cannot be generalized that one certain environmental condition leads to a certain kind of cell wall (König et al., 2007), this is true for halophilic archaea in particular and all other archaea in general. With the increasing number of archaea, which were described to be surrounded by two membranes like ultra-small ARMAN cells, *Methanomassiliicoccus luminyensis* or the SM1 euryarchaeon, particular attention should be paid to this topic. For example the SM1 euryarchaeon was already known for more than 10 years, without having data about its cell wall structure (Rudolph et al., 2001).

Interestingly, a common feature of all archaea that posses a double membrane cell wall architecture is that they are closely interacting with other organisms (archaea, bacteria, eukaryotes), as already mentioned by Perras et al. (2014), and that they are difficult to cultivate or even not cultivatable at all. At this point, it can still be discussed if the S-layer (Albers and Meyer, 2011) or an OM is the more archaic cell wall compound. With recently developed and refined isolation and preparation methods, ongoing investigations should be able to shed light on further structural and biochemical features of archaeal outermost cellular membranes. Especially the localization of protein complexes like the ATPase in the cytoplasmic membrane like in Gram-negative bacteria or in the outermost cellular membrane like in *Ignicoccus hospitalis* (Küper et al., 2010) seems to be crucial in this concern.

## Conflict of Interest Statement

The author declares that the research was conducted in the absence of any commercial or financial relationships that could be construed as a potential conflict of interest.

## References

[B1] Akça E. Claus H. Schultz N. Karbach G. Schlott B. Debaerdemaeker T. (2002). Genes and derived amino acid sequences of S-layer proteins from mesophilic, thermophilic and extremely thermophilic methanococci. *Extremophiles* 6 351–358 10.1007/s00792-001-0264-1 12382110

[B2] Albers S.-V. Meyer B. H. (2011). The archaeal cell envelope. *Nat. Rev. Microbiol.* 9 414–426 10.1038/nrmicro2576 21572458

[B3] Baumeister W. Lembcke G. (1992). Structural features of archaebacterial cell envelopes. *J. Bioenerg. Biomembr.* 24 567–575 10.1007/BF00762349 1459988

[B4] Beveridge T. J. Graham L. L. (1991). Surface layers of bacteria. *Microbiol. Rev.* 55 684–705. 1723487 10.1128/mr.55.4.684-705.1991 PMC372843

[B5] Beveridge T. J. Patel G. B. Harris B. J. Sprott G. D. (1986). The ultrastructure of *Methanothrix concilii*, a mesophilic aceticlastic methanogen. *Can. J. Microbiol.* 32 703–710 10.1139/m86-128

[B6] Beveridge T. J. Stewart M. Doyle R. J. Sprott G. D. (1985). Unusual stability of the *Methanospirillum hungatei* sheath. *J. Bacteriol.* 162 728–737. 3988711 10.1128/jb.162.2.728-737.1985 PMC218911

[B7] Bolhuis H. Palm P. Wende A. Falb M. Rampp M. Rodriguez-Valera F. (2006). The genome of the square archaeon *Haloquadratum walsbyi*: life at the limits of water activity. *BMC Genomics* 7:169 10.1186/1471-2164-7-169 PMC1544339 16820047

[B8] Brown A. D. Cho K. J. (1970). The cell walls of extremely halophilic cocci. Gram-positive bacteria lacking muramic acid. *J. Gen. Microbiol.* 62 267–270 10.1099/00221287-62-2-267 5493598

[B9] Burghardt T. Näther D. J. Junglas B. Huber H. Rachel R. (2007). The dominating outer membrane protein of the hyperthermophilic archaeum *Ignicoccus hospitalis*: a novel pore-forming complex. *Mol. Microbiol.* 63 166–176 10.1111/j.1365-2958.2006.05509.x 17163971

[B10] Burns D. G. Janssen P. H. Itoh T. Kamekura M. Li Z. Jensen G. (2007). *Haloquadratum walsbyi* gen. nov., sp. nov., the square haloarchaeon of Walsby, isolated from saltern crystallizers in Australia and Spain. *Int. J. Syst. Evol. Microbiol.* 57 387–392 10.1099/ijs.0.64690-0 17267984

[B11] Claus H. Akça E. Debaerdemaeker T. Evrard C. Declercq J. P. Harris J. R. (2005). Molecular organization of selected prokaryotic S-layer proteins. *Can. J. Microbiol.* 51 731–743 10.1139/w05-093 16391651

[B12] Claus H. Akça E. Debaerdemaeker T. Evrard C. Declercq J. P. König H. (2002). Primary structure of selected archaeal mesophilic and extremely thermophilic outer surface proteins. *Syst. Appl. Microbiol.* 25 3–12 10.1078/0723-2020-00100 12086185

[B13] Claus H. Akça E. Schultz N. Karbach G. Schlott B. Debaerdemaeker T. (2001). “Surface (glyco-)proteins: primary structure and crystallization under microgravity conditions,” in *Proceedings of the First European Workshop on Exo-/Astro-Biology, ESA SP-496* Frascati 806–809.

[B14] Comolli L. R. Baker B. J. Downing K. H. Soegerist C. E. Banfield J. F. (2009). Three-dimensional analysis of the structure and ecology of a novel, ultra-small archaeon. *ISME J.* 3 159–167 10.1038/ismej.2008.99 18946497

[B15] Dridi B. Fardeau M.-L. Ollivier B. Raoult D. Drancourt M. (2012). *Methanomassiliicoccus luminyensis* gen. nov., sp. nov., a methanogenic archaeon isolated from human faeces. *Int. J. Syst. Evol. Microbiol.* 62 1902–1907 10.1099/ijs.0.033712-0 22859731

[B16] Eichler J. (2003). Facing extremes: archaeal surface-layer (glyco)proteins. *Microbiology* 149 3347–3351 10.1099/mic.0.26591-0 14663068

[B17] Engelhardt H. (2007a). Are S-layers exoskeletons? The basic function of protein surface layers revisited. *J. Struct. Biol.* 160 115–124 10.1016/j.jsb.2007.08.003 17889557

[B18] Engelhardt H. (2007b). Mechanism of osmoprotection by archaeal S-layers: a theoretical study. *J. Struct. Biol.* 160 190–199 10.1016/j.jsb.2007.08.004 17888677

[B19] Firtel M. Southam G. Harauz G. Beveridge T. J. (1993). Characterization of the cell wall of the sheathed methanogen *Methanospirillum hungatei* Gp1 as an S-layer. *J. Bacteriol.* 175 7550–7560. 8244924 10.1128/jb.175.23.7550-7560.1993 PMC206911

[B20] Haney P. J. Badger J. H. Buldak G. L. Reich C. I. Woese C. R. Olsen G. J. (1999). Thermal adaption analyzed by comparison of protein sequences from mesophilic and extremely thermophilic *Methanococcus* species. *Proc. Natl. Acad. Sci. U.S.A.* 36 3578–3583 10.1073/pnas.96.7.3578 10097079 PMC22336

[B21] Houwink A. L. (1956). Flagella, gas vacuoles and cell-wall structure in *Halobacterium halobium*; an electron microscope study. *J. Gen. Microbiol.* 15 146–150 10.1099/00221287-15-1-146 13357722

[B22] Huber H. Hohn M. J. Rachel R. Fuchs T. Wimmer V. C. Stetter K. O. (2002). A new phylum of archaea represented by a nanosized hyperthermophilic symbiont. *Nature* 417 63–67 10.1038/417063a 11986665

[B23] Huber H. Küper U. Daxer S. Rachel R. (2012). The unusual cell biology of the hyperthermophilic Crenarchaeon *Ignicoccus hospitalis*. *Antonie Van Leeuwenhoek* 102 203–219 10.1007/s10482-012-9748-5 22653377

[B24] Jahn U. Summons R. Sturt H. Grossjean E. Huber H. (2004). Composition and source of the lipids of *Nanoarchaeum equitans* and their origin in the cytoplasmic membrane of its host Ignicoccus sp. KIN4I. *Arch. Microbiol.* 182 404–413 10.1007/s00203-004-0725-x 15492905

[B25] Jarrell K. G. Ding Y. Meyer B. H. Albers S.-V. Kaminski L. Eichler J. (2014). N-linked glycosylation in archaea: a structural, functional, and genetic analysis. *Microbiol. Mol. Biol. Rev.* 78 304–341 10.1128/MMBR.00052-13 24847024 PMC4054257

[B26] Junglas B. Briegel A. Burghardt T. Walther P. Wirth R. Huber H. (2008). *Ignicoccus hospitalis* and *Nanoarchaeum equitans*: ultrastructure, cell-cell interaction, and 3D reconstruction from serial sections of freeze-substituted cells and by electron cryotomography. *Arch. Microbiol.* 190 395–408 10.1007/s00203-008-0402-6 18622597 PMC2755780

[B27] Kandler O. Hippe H. (1977). Lack of peptidoglycan in the cell walls of *Methanosarcina barkeri*. *Arch. Microbiol.* 113 57–60 10.1007/BF00428580 889387

[B28] Kandler O. König H. (1978). Chemical composition of the peptidoglycan-free cell walls of methanogenic bacteria. *Arch. Microbiol.* 118 141–152 10.1007/BF00415722 697504

[B29] Kandler O. König H. (1985). “Cell envelopes of archaebacteria,” in *The Bacteria. A Treatise on Structure and Function. Archaebacteria* Vol. VIII Woese C. R. Wolfe R. S. (New York: Academic Press) 413–457.

[B30] Kandler O. König H. (1993). “Cell envelopes of archaea: structure and chemistry,” in *The Biochemistry of Archaea (Archaebacteria)*, edsKates M. Kusher D. Matheson A. T. (Amsterdam: Elsevier Science Publication) 223–333.

[B31] Kandler O. König H. (1998). Cell wall polymers in archaea (Archaebacteria). *Cell Mol. Life Sci.* 54 305–308 10.1007/s000180050156 9614965 PMC11147200

[B32] Kärcher U. Schröder H. Haslinger E. Allmeier G. Schreiner R. Wieland F. (1993). Primary structure of the heterosaccharide of the surface glycoprotein of *Methanothermus fervidus*. *J. Biol. Chem.* 268 26821–26826. 8262914

[B33] Kates M. (1978). The phytanyl ether-linked polar lipids and isoprenoid neutral lipids of extremely halophilic bacteria. *Prog. Chem. Fats Other Lipids* 15 301–342 10.1016/0079-6832(77)90011-8 358256

[B34] Kates M. (1992). Archaebacterial lipids: structure, biosynthesis and function. *Biochem. Soc. Symp.* 58 51–72. 1445410

[B35] Kjellen L. Lindahl U. (1991). Proteoglycans: structures and interactions. *Annu. Rev. Biochem.* 60 443–475 10.1146/annurev.bi.60.070191.002303 1883201

[B36] Klingl A. Flechsler J. Heimerl T. Rachel R. (2013). Archaeal Cells. In: eLS. John Wiley & Sons, Ltd: Chichester.

[B37] Klingl A. Moissl-Eichinger C. Wanner G. Zweck J. Huber H. Thomm M. (2011). Analysis of the surface proteins of *Acidithiobacillus ferrooxidans* strain SP5/1 and the new, pyrite-oxidizing *Acidithiobacillus* isolate HV2/2, and their possible involvement in pyrite oxidation. *Arch. Microbiol.* 193 867–882 10.1007/s00203-011-0720-y 21698546

[B38] König H. Hartmann E. Kärcher U. (1994). Pathways and principles of the biosynthesis of methanobacterial cell wall polymers. *Syst. Appl. Microbiol.* 16 510–517 10.1016/S0723-2020(11)80320-6

[B39] König H. Rachel R. Claus H. (2007). “Proteinaceous surface layers of archaea: ultrastructure and biochemistry,” in *Archaea: Molecular and Cell Biology*, ed.Cavicchioli R. (Washington, DC: American Society of Microbiology Press) 315–340.

[B40] König H. Stetter K. O. (1986). Studies on archaebacterial S-layers. *Syst. Appl. Microbiol.* 7 300–309 10.1016/S0723-2020(86)80023-6

[B41] Koval S. Jarrell K. (1987). Ultrastructure and biochemistry of the cell wall of *Methanococcus voltae*. *J. Bacteriol.* 169 1298–1306. 3818546 10.1128/jb.169.3.1298-1306.1987 PMC211934

[B42] Kreisl P. Kandler O. (1986). Chemical structure of the cell wall polymer of *Methanosarcina*. *Syst. Appl. Microbiol.* 7 293–299 10.1016/S0723-2020(86)80022-4

[B43] Küper U. Meyer C. Müller V. Rachel R. Huber H. (2010). Energized outer membrane and spatial separation of metabolic processes in the hyperthermophilic archaeon *Ignicoccus hospitalis*. *Proc. Natl. Acad. Sci. U.S.A.* 107 3152–3156 10.1073/pnas.0911711107 20133662 PMC2840320

[B44] Kurr M. Huber R. König H. Jannasch H. W. Fricke H. Trincone A. (1991). *Methanopyrus kandleri*, gen. and sp. nov. represents a novel group of hyperthermophilic methanogens, growing at 110° C. *Arch. Microbiol.* 156 239–247 10.1007/BF00262992

[B45] Lechner J. Sumper M. (1987). The primary structure of a procaryotic glycoprotein. Cloning and sequencing of the cell surface glycoprotein gene of halobacteria. *J. Biol. Chem.* 262 9724–9729. 3036870

[B46] Mescher M. F. Strominger J. L. (1976a). Purification and characterisation of a prokaryotic glycoprotein from the cell envelope of *Halobacterium salinarium*. *J. Biol. Chem.* 251 2005–2014. 1270419

[B47] Mescher M. F. Strominger J. L. (1976b). Structural (shape-maintaining) role of the cell surface glycoprotein of *Halobacterium salinarium*. *Proc. Natl. Acad. Sci. U.S.A.* 73 2687–2691 10.1073/pnas.73.8.2687 1066681 PMC430713

[B48] Messner P. Sleytr U. B. (1992). Crystalline bacterial cell-surface layers. *Adv. Microbial. Physiol.* 33 213–274 10.1016/S0065-2911(08)60218-0 1636510

[B49] Näther D. J. Rachel R. (2004). The outer membrane of the hyperthermophilic archaeon *Ignicoccus*: dynamics, ultrastructure and composition. *Biochem. Soc. Trans.* 32 199–203 10.1042/BST0320199 15046571

[B50] Niemetz R. Kärcher U. Kandler O. Tindall B. König H. (1997). The cell wall polymer of the extremely halophilic archaeon *Natronococcus occultus*. *Eur. J. Biochem.* 249 905–911 10.1111/j.1432-1033.1997.00905.x 9395342

[B51] Nußer E. König H. (1987). S-layer studies on three species of *Methanococcus* living at different temperatures. *Can. J. Microbiol.* 33 256–261 10.1139/m87-043

[B52] Perras A. K. Wanner G. Klingl A. Mora M. Auerbach A. K. Heinz V. (2014). Grappling archaea: ultrastructural analyses of an uncultivated, cold-loving archaeon, and its biofilm. *Front. Microbiol.* 5:397 10.3389/fmicb.2014.00397 PMC4122167 25140167

[B53] Rachel R. Meyer C. Klingl A. Gürster S. Heimerl T. Wasserburger N. (2010). Analysis of the ultrastructure of archaea by electron microscopy. *Methods Cell Biol.* 96 47–69 10.1016/S0091-679X(10)96003-2 20869518

[B54] Rachel R. Wyschkony I. Riehl S. Huber H. (2002). The ultrastructure of Ignicoccus: evidence for a novel outer membrane and for intracellular vesicle budding in an archaeon. *Archaea* 1 9–18 10.1155/2002/307480 15803654 PMC2685547

[B55] Rigel N. W. Silhavy T. J. (2012). Making a beta-barrel: assembly of outer membrane proteins in Gram-negative bacteria. *Curr. Opin. Microbiol.* 15 189–193 10.1016/j.mib.2011.12.007 22221898 PMC3320693

[B56] Rudolph C. Wanner G. Huber R. (2001). Natural communities of novel archaea and bacteria growing in cold sulfurous springs with a string-of-pearls-like morphology. *Appl. Environ. Microbiol.* 67 2336–2344 10.1128/AEM.67.5.2336-2344.2001 11319120 PMC92875

[B57] Ruiz N. Kahne D. Silhavy T. J. (2006). Advances in understanding bacterial outer-membrane biogenesis. *Nat. Rev. Microbiol.* 4 57–66 10.1038/nrmicro1322 16357861

[B58] Shaw P. J. Hills G. J. Henwood J. A. Harris J. E. Archer D. B. (1985). Three-dimensional architecture of the cell sheath and septa of *Methanospirillum hungatei*. *J. Bacteriol.* 161 750–757. 3968038 10.1128/jb.161.2.750-757.1985 PMC214946

[B59] Sleytr U. B. Messner P. Pum D. Sára M. (1988). “Crystalline bacterial cell surface layers,” in *Proceedings of EMBO Workshop on Crystalline Bacterial Surface Layers*. Berlin: Springer Verlag.

[B60] Sleytr U. B. Messner P. Pum D. Sára M. (1999). Crystalline bacterial cell surface layers (S-layers): from supramolecular cell structure to biomimetics and nanotechnology. *Angew. Chem. Int. Ed. Engl.* 38 1034–1054 10.1002/(SICI)1521-3773(19990419)38:8<1034::AID-ANIE1034>3.0.CO;2-# 25138491

[B61] Sprott G. D. Colvin J. R. Mckellar R. C. (1979). Spheroblasts of *Methanospirillum hungatai* formed upon treatment with dithiothreitol. *Can. J. Microbiol.* 25 730–738 10.1139/m79-106 38895

[B62] Sprott G. D. McKellar R. C. (1980). Composition and properties of the cell wall of *Methanospirillum hungatei*. *Can. J. Microbiol.* 26 115–120 10.1139/m80-017 7407700

[B63] Stetter K. O. Thomm M. Winter J. Wildgruber G. Huber H. Zillig W. (1981). *Methanothermus fervidus*, sp. nov., a novel extremely thermophilic methanogen isolated from an Icelandic hot spring. *Zbl. Bakt. Hyg. I. Abt. Orig.* C2 166–178.

[B64] Stieglmeier M. Klingl A. Rittmann S. Alves R. E. Melcher M. Leisch N. (2014). *Nitrososphaera viennensis* sp. nov., an aerobic and mesophilic ammonia oxidizing archaeon from soil and member of the novel archaeal phylum Thaumarchaeota. *Int. J. Syst. Evol. Microbiol.* 64 2738–2752 10.1099/ijs.0.063172-0 24907263 PMC4129164

[B65] Sumper M. Berg E. Mengele R. Strobel I. (1990). Primary structure and glycosylation of the S-layer protein of *Haloferax volcanii*. *J. Bacteriol.* 172 7111–7118. 2123862 10.1128/jb.172.12.7111-7118.1990 PMC210834

[B66] Sumper M. Wieland F. T. (1995). “Bacterial glycoproteins,” in *Glycoproteins*, eds Montreuil J. Vliegenthart J. F. G. Schachter H. (Amsterdam: Elsevier) 455–473.

[B67] Tokuda H. (2009). Biogenesis of outer membranes in Gram-negative bacteria. *Biosci. Biotechnol. Biochem.* 73 465–473 10.1271/bbb.80778 19270402

[B68] Veith A. Klingl A. Zolghadr B. Lauber K. Mentele R. Lottspeich F. (2009). *Acidianus*, *Sulfolobus* and *Metallosphaera* surface layers: structure, composition and gene expression. *Mol. Microbiol.* 73 58–72 10.1111/j.1365-2958.2009.06746.x 19522740

[B69] Wildhaber I. Baumeister W. (1987). The cell envelope of *Thermoproteus tenax*: three-dimensional structure of the surface layer and its role in shape maintenance. *EMBO J.* 6 1475–1480. 16453767 10.1002/j.1460-2075.1987.tb02389.x PMC553954

[B70] Yurist-Doutsch S. Chaban B. VanDyke D. J. Jarrell K. F. Eichler J. (2008). Sweet to the extreme: protein glycosylation in archaea. *Mol. Microbiol.* 68 1079–1084 10.1111/j.1365-2958.2008.06224.x 18476920

[B71] Zehnder A. J. B. Huser B. A. Brock T. B. Wuhrmann K. (1980). Characterization of an acetate dacarboxylating, non hydrogen oxidizing methane bacterium. *Arch. Microbiol.* 124 1–11 10.1007/BF00407022 6769415

[B72] Zeikus J. G. Bowen V. G. (1975). Fine structure of *Methanospirillum hungatei*. *J. Bacteriol*. 121 373–380. 46863 10.1128/jb.121.1.373-380.1975 PMC285652

